# Immunogenicity of a Fusion Protein Containing Immunodominant Epitopes of Ag85C, MPT51, and HspX from *Mycobacterium tuberculosis* in Mice and Active TB Infection

**DOI:** 10.1371/journal.pone.0047781

**Published:** 2012-10-25

**Authors:** Eduardo Martins de Sousa, Adeliane Castro da Costa, Monalisa Martins Trentini, João Alves de Araújo Filho, André Kipnis, Ana Paula Junqueira-Kipnis

**Affiliations:** Instituto de Patologia Tropical e Saúde Pública, Universidade Federal de Goiás, Goiânia, Goiás, Brazil; University of Delhi, India

## Abstract

Tuberculosis (TB) remains a major global health problem. The only vaccine against tuberculosis, attenuated *Mycobacterium bovis* Bacillus Calmette-Guerin (BCG), has demonstrated relatively low efficacy and does not provide satisfactory protection against the disease in adults. More effective vaccines and better therapies are urgently needed to reduce the global spread of TB. This study evaluated the immunogenicity of a recombinant *M. tuberculosis* Ag85C-MPT51-HspX fusion protein (CMX) in mice and individuals with active tuberculosis. BALB/c mice were immunized with the CMX protein liposome-encapsulated with CpG DNA or with CpGDNA liposome-encapsulated, liposome or saline as negative controls. The immunization produced high levels of anti-CMX -specific IgG1 and IgG2a antibodies and induced an increase in the relative and absolute numbers of specific TCD4 IFN-γ^+^ and TNF-α^+^ cells in the spleen. Sera from a cohort of individuals with active tuberculosis contained higher levels of IgG and IgM that recognized CMX when compared to healthy individuals. In conclusion, this protein was shown to be immunogenic both in mice and humans.

## Introduction

Tuberculosis (TB) is an infectious disease arousing great public health concerns [1]; there were 1.1 million deaths from TB and 8.8 million new cases in 2010, according to WHO [2]. The epidemic of tuberculosis associated with HIV co-infection has increased the incidence of TB considerably, especially in developing countries. The main obstacles to controlling TB worldwide are multidrug resistance, the absence of concise diagnostic methods, and variations in the protective effects of the BCG vaccination.

In some developing countries, such as Brazil, TB is primarily diagnosed in the clinic by radiological evaluation of the lungs and other tests, such as the tuberculin skin test (TST) and detection of acid-fast bacilli in sputum samples by direct staining or by microbiological culture. However, the currently used tests have not been effective in further reducing the incidence of TB in these countries. Brazil is 19^th^ out of the 22 countries responsible for 80% of the TB cases worldwide. According to the Ministry of Health, seventy one thousand new cases of tuberculosis were diagnosed in Brazil in 2010, and consequently, TB is considered endemic in Brazil. In Brazil, 86.7% of the pulmonary TB cases are diagnosed by acid-fast bacilli detection in sputum samples [3]. Worldwide estimates indicate that about two billion people have latent infections, and 10% of these individuals will develop active disease [4].

New tests to diagnose TB and latent TB infections (LTBI) have been developed based on the evaluation of the specific cellular immune response against *Mycobacterium tuberculosis* (Mtb), the causative agent of TB. These tests evaluate the production of IFN-γ by cells stimulated with two specific Mtb antigens (ESAT-6 and CFP-10), which are absent from the BCG vaccine strains and most environmental mycobacteria [5]. The interferon-γ release assay (IGRA) has improved the capacity to diagnose TB and LTBI over the tuberculin skin test (TST), as a consequence of the increased specificity [6]. In a TB endemic area, where most of the population has already been in contact with Mtb, an IGRA response may reflect the increased bacterial replication associated with the development of active TB [7]. Some advantages of the IGRA for the diagnosis of TB and LTBI include the following: the test requires only one laboratory visit, the results are rapid, and the criteria for interpreting the results are less subjective than for the TST. The main disadvantages of the IGRA include the requirements for laboratory infrastructure and skilled personnel to perform the tests. Another recently developed approach to improve TB diagnosis is the Xpert MTB/RIF test, a molecular detection test that can concomitantly detect Mtb DNA in the sputum samples and mutations in the major genes responsible for Rifampin (RIF) resistance [8]. The rapid availability of results provided by the Xpert MTB/RIF test, together with the analysis of resistance to chemotherapeutics provides another choice for the detection of the bacilli in sputum samples from patients. However, the ability of this test to exclude the disease is not optimal because the sensitivity for the detection of bacilli in extrapulmonary TB is less than 50% [9]. In addition, the use of the Xpert MTB/RIF test for the diagnosis of TB in developing countries, such as places in Africa where most people are co-infected with TB-HIV, is highly cost-effective [10]. Therefore, developing a reliable diagnostic method to effectively discriminate between TB and LTBI, which can rapidly generate results and is easy to perform at a low cost, is of utmost importance.

An alternative to improving the immunological-based diagnostic tests is the use of a source of recombinant Mtb proteins produced under different growth conditions that can be detected *in vitro* or during different stages of *in vivo* infection. Thus, the association of proteins recognized at different stages of human infection could efficiently identify individuals infected with TB at different stages of disease development. Our group and others have demonstrated that the antigens MPT51 and HspX are recognized by the immune systems of individuals with active and latent TB infections [11]–[13]. Analyzing both the humoral and cellular immune response to MPT51, for example, a discrimination between patients with active TB versus healthy individuals was observed, while the humoral immune response to HspX seems to be associated with latency. The MPT51 protein belongs to the family of α/ß non-catalytic hydrolases that are likely to be involved in bacterial adhesion to the extracellular matrix. HspX, or alpha-crystallin protein, is expressed preferentially during the lag phase of the Mtb infection [14], and has also been demonstrated in bacilli that are confined within granulomas or macrophages, under conditions of limited energy sources and oxygen deprivation. Furthermore, HspX epitopes are also recognized by CD4 and CD8 T cells from patients with active TB [15].

The Ag85 complex is a family of 30–32 kDa proteins (Ag85A, Ag85B, Ag85C) secreted by Mtb. Antigen 85 is involved in the formation of the mycobacteria cell wall by catalyzing the transfer of mycolates, leading to the synthesis of trehalose dimycolate (TDM), which is an important mycobacteria virulence factor [16]. Ag85A is a major component of the Ag85 complex, which confers protection when used as subunit vaccine in mice and guinea pigs challenged with Mtb [17]. Ag85 has been shown to be crucial for Mtb growth in macrophage cell lines and is also recognized at the early stages of experimental TB infection and by T cells from TB patients [18]–[19]. Ag85C has a specific mycolyltransferase activity during cell wall biosynthesis that is distinct from Ag85A and Ag85B [20]. Ag85C can be recognized by T CD4 and CD8 cells from individuals with active TB. Moreover, sera from children with TB infections distinctly recognize Ag85C, despite exhibiting negative acid fast staining smears and Mtb cultures [21]–[22].

In the present study, we have assessed whether a recombinant fusion protein (CMX), containing immunodominant epitopes of the Ag85C, MPT51, and HspX proteins from *M. tuberculosis,* was immunogenic in mice, and could be specifically recognized by the humoral immune response in individuals with active TB.

## Materials and Methods

### Ethics Statement

This study was carried out in strict accordance with the recommendations in the Guide for the Care and Use of Laboratory Animals of Sociedade Brasileira de Animais de Laboratorio and COBEA- Colégio Brasileiro de Experimentação Animal. The protocol for this work was approved by the Committee on the Ethics of Animal Experiments of the University Federal de Goias (Permit Number: 229/11).

The ethical requirement for research on human subjects was obtained. The Ethical Committee from Universidade Federal de Goias (CoEP - Comitê de Etica em Pesquisa da Universidade Federal de Goias) approved this work and the informed consent using the CNS196/96 that follows the Declaration of Helsinki. All patients provided written informed consent for the collection of samples and subsequent analysis. (Permit number: 002/05).

### Collection of Blood Serum

Blood samples were collected from individual TB patients at the Anuar Auad Hospital (Anuar Auad Tropical Disease Hospital, HDTAA) in Goiânia, Goiás and the Reference Center for Diagnosis and Therapy, Brazil between March 2005 and March 2006.

Forty-three sera samples from healthy controls, involving twenty-two samples from individuals vaccinated with BCG and twenty-one samples from patients of unknown health status, were included in this study. Fifty-three samples from individuals with active TB were analyzed; among these patients, forty-seven were TB-positive by smear microscopy. The details regarding the study population are shown in Table 1.

**Table 1:** Clinical and epidemiological characteristics of the individuals used in the study.

Group	Healthy Controls	Active TB
Gender (Male/Female)	32/11	40/13
Average Age (Variance)	35,2: (19–73)	41,5: (20–73)
BCG (Vaccinated/NI^a^)	22/21	39/14
AFB^c^ (Positive/Negative/NI^a^)	−	47/04/02
Culture (Positive/Negative/NR^b^)	−	16/01/36

a No information.

b Not tested.

c Acid-fast bacilli smear.

### Criteria for Inclusion and Exclusion

The volunteers were selected independently of sex, age, and residency in the state of Goias, Brazil (Table 1).

The patients were enrolled at two Public Health Units of Goiás, the Dr. Anuar Auad Tropical Diseases Hospital and the Reference Center for Diagnosis and Therapy, between March 2005 and March 2006. The inclusion criteria for the subjects included a diagnosis of TB according to the Brazilian Ministry of Health guidelines [23], which considers patients who present with clinical manifestations, such as fever and productive cough, for more than three weeks, a positive acid-fast smear or Mtb culture, and a chest x-ray suggestive for the disease as having active TB infection. Individuals with other chronic diseases, immunosuppressed individuals, such as those with HIV infections or using suppressive drugs (steroids), pregnant women or those without a confirmed diagnosis, or individuals under the age of 18 were excluded.

The control group was randomly selected among healthy volunteers in the community. They were residents of the state of Goiás who met the following criteria: not known to have had prior contact with an indexed case of TB, TST non-reactive, HIV-seronegative and without manifestations of chronic or parasitic disease. We excluded individuals using immunosuppressive drugs and pregnant women. The control group was paired according to sex and age (±6 years) with patients with active tuberculosis.

The clinical and epidemiological data about the individuals with active TB were obtained after review of their clinical charts. The data from healthy controls were obtained upon recruitment.

### PCR Amplification, Cloning and Sequencing

The nucleotide sequences corresponding to epitopes 85 to 159 bp of Ag85C (RV0129c) and 91 to 112 bp of MPT51 (RV3803c) and the entire of HspX gene (RV2031c) were PCR amplified from the *M. tuberculosis* H37Rv genome using primers (Table S1) designed to facilitate posterior cloning and fusion construction.

The PCR products of the Ag85C, MPT51 and HspX genes were individually cloned into the pGEM-T easy vector (PROMEGA®). Each recombinant plasmid was digested with the appropriate enzymes (as listed in Table S1). The digested genes where purified from agarose gels and used in a 15 µL ligation reaction. Two microliters of the ligation reaction was used in a new PCR containing primers for the amino portion of Ag85C and the carboxy portion of HspX to amplify the ligated fusion gene. The PCR product corresponding to the fusion gene was purified from an agarose gel and cloned into the pGEM-T easy vector (Fig. S1).

### Cloning into the pET23a Vector and Expression of the Fusion Gene

The recombinant pGEM-T vector was digested with *HindIII* and *BamHI* to release the fusion gene. After agarose gel elution, the digested fusion gene was ligated into the pET23a expression vector (Novagen Biosciences), which had previously been digested with the same enzymes. The recombinant pET23a construct containing the fusion gene (pET23a/CMX) (Fig. S2) had its sequence checked with a BI 3130 capillary DNA sequencer (Applied Biosystems, CA) and was then inserted into the expression host *E. coli* BL21 (DE3) pLysS. Bacteria containing the recombinant expression vector were grown in Luria–Bertani (LB) broth supplemented with ampicillin (100 µg/mL) and chloramphenicol (20 µg/mL). When the bacterial cells reached mid-log growth (OD_600_ measurements of 0.4–0.6), the expression of the fusion protein was induced by the addition of isopropyl-beta-D-thiogalactopyranoside (IPTG) to a final concentration of 1 mM, and the incubation continued at 37°C for 4 h. Similar culture conditions without the addition of IPTG were used to control for protein induction. The bacterial cells were collected by centrifugation (10,000 g for 5 min), and the cell pellets were suspended in sodium dodecyl sulfate polyacrylamide gel electrophoresis (SDS-PAGE) sample buffer to analyze the expression of the recombinant proteins. *E. coli* BL21 (DE3) pLysS carrying the empty pET23a vector was used as a negative control.

### SDS-PAGE and Western Blotting

Western blotting was performed based on the identification of the histidine tag expressed at the carboxyl end of the recombinant fusion protein, provided by the pET23a vector. Accordingly, the SDS-PAGE separated proteins were electrotransferred onto a nitrocellulose membrane and incubated with a 1∶2000 dilution of horseradish peroxidase (HRP)-conjugated anti-penta-His antibody (Sigma-Aldrich®). The presence of histidine tagged proteins was detected by incubation with diaminobenzidine (DAB) substrate (Roche, Germany).

### Purification of Recombinant Fusion Protein

The recombinant fusion protein was purified based on the presence of the 6× His-tag at the N-terminus using Ni-NTA affinity columns (Qiagen, Germany) under denaturing conditions. To purify the protein, a large-scale preparation of *E. coli* cells expressing the recombinant fusion protein (*pET23a/CMX*) was grown in 1 L of LB. The final bacterial cell pellet was resuspended in 8 mL of denaturing lysis buffer (6 M guanidine HCl, 20 mM sodium phosphate, pH 7.8, 500 Mm NaCl). After the lysates were loaded on the Ni-NTA column and extensively washed, the recombinant fusion protein was eluted with elution buffer (8 M urea, 20 mM Na_2_HPO_4_, 500 mM NaCl, 500 mM imidazole, pH 4.0). The purity of the recombinant protein was then evaluated by SDS-PAGE. The protein concentration was determined by the Bradford assay (Bio-Rad® Protein Assay) using bovine serum albumin as a standard, according to the manufacturer’s instructions.

### Indirect ELISA for Detection of Human Serum IgM and IgG

Ninety-six well polystyrene plates (Costar) were adsorbed with the fusion protein (10 µg/mL) diluted in 0.015 M carbonate-bicarbonate buffer, (pH 9.6). After incubation for 18 h at 4–8°C, the plates were blocked using 100 mL of blocking solution, composed of carbonate-bicarbonate buffer and 1% skim milk, and again incubated for two hours at 37°C. Fifty microliters per well of the serum samples from pulmonary TB patients and healthy controls diluted 1∶40 in PBS containing 0.06% skim milk were added and incubated at 37°C for 2 h. After incubation, the plates were washed six times with PBS containing 0.05% Tween 20, and 50 µL of human IgM and IgG conjugated to horseradish peroxidase (Sigma Aldrich®) were added at concentrations of 1∶2,500 and 1∶10,000, respectively, diluted in PBS containing 0.06% skim milk and incubated for one hour at 37°C. The washes were repeated, and 50 µL of citrate-phosphate buffer containing OPD (1 mg/mL) and 20 µL of H_2_O_2_ were added and incubated for 15 min at room temperature in the dark. After this period, 4 N H_2_SO_4_ was added to block the enzymatic reaction. The samples were read using an ELISA reader (Multiskan Plus) at 492 ηm. The samples were tested in duplicate, and the mean optical density was expressed as the absorbance.

### Animals

Specific pathogen-free 4–6 week old BALB/c female mice from the animal care facility of the Institute of Tropical Pathology and Public Health at UFG were maintained in isolators in class 2 biosafety level (BL2) cabinets with water and food provided ad libitum. The temperature was maintained between 20–24°C with the relative humidity set between 40–70%, and 12 h light/dark cycles. The use of mice was conducted in accordance with the guidelines of the Brazilian Society of Animal Science Laboratory (SBCAL/COBEA). The protocol was approved by the Committee on the Ethics of Animal Experiments of the Universidade Federal de Goiás, (Permit Number: 229/11).

### Immunization

Mice were immunized three times with an interval of 15 days between immunizations. Ten micrograms of the CMX fusion protein combined with 20 µg of CpG DNA (ODN 1826, InvivoGen®) liposome-encapsulated in a volume of 100 µL were subcutaneously (sc) injected into the neck. The control groups received 100 µL of the combination of 20 µg liposome-encapsulated CpG DNA or 100 µL of liposome alone. The liposome vaccine formulations were prepared by the Laboratory of Pharmaceutical Technology (FARMATEC-UFG), using the lipid film hydration (HFL) method. Negative control mice received 100 µL of sterile saline.

### Serum Collection

Serum samples were obtained 15 days after the third immunization. The samples were incubated for one hour at 37°C, centrifuged at 1,200 g at 4°C for 15 min for serum separation, and stored at −20°C.

### ELISA of Mouse Samples

Ninety-six well polystyrene plates (Nunc®) were initially coated with the CMX fusion protein or recombinant MPT51 or recombinant HspX or Ag85C (5 µg/mL) diluted in 0.05 M sodium carbonate/bicarbonate buffer (pH 9.6) and incubated at 4°C for 16 h. Subsequently, the plate was washed and incubated for one h at 37°C with sodium carbonate buffer (PBS) containing 1% skim milk. The samples were diluted 1∶1,000, added to the wells and incubated for 2 h at 37°C. After several washes, 1∶5,000 diluted biotin-conjugated antibody was added to the plates (anti-IgG1 and anti-mouse IgG2a; Pharmingen®). The plates were incubated for 1 h at 37°C, after which time streptavidin peroxidase diluted 1∶1,000 was added, and the plates were again incubated for 1 h at 37°C. The reaction was then developed with citrate phosphate buffer containing ortho-phenylenediamine (OPD) and hydrogen peroxide and stopped after 15 min by adding 4 N sulfuric acid. The absorbance at 492 ηm was read in an ELISA reader (Labsystems Multiskan Thermo®). Between each step, the plates were washed six times with PBS containing 0.05% Tween 20.

### Evaluation of the Cellular and Cytokine Components of the Spleens of Immunized Mice

Thirty days after the final immunization, the mice were sacrificed. The spleen was removed aseptically, and the cells were separated using sterile tweezers. A cell suspension was prepared, and the erythrocytes were lysed with Gey’s solution. The cells were washed in RPMI 1640 supplemented with 10% fetal bovine serum. The cell concentration was adjusted to 1×10^6^ cells/mL, and the cells were plated in a 96-well plate. The splenocytes were stimulated with ConA (10 µg/mL) or CMX (10 µg/mL) or not stimulated. After incubation for 4 h at 37°C and 5% CO_2_ with monensin (eBioscience), the cells were harvested for intracellular cytokine staining. Thereafter, the cells were stained with CD4, IFN-γ and TNF-α using the BD Cytofix/Cytoperm kit according to the manufacturer’s instructions. The cells were fixed with PBS 0.05% sodium azide. The acquisition was performed using a BD Biosciences FACSCanto II flow cytometer, and the data were analyzed using BD FACSDiva software. For each sample, 100 thousands events were acquired.

### Statistical Analysis

The results were tabulated using Excel (version 2007) and Prism 4 software (GraphPad Software 4.0). The differences between groups were assessed using the two-tailed Student’s t-test after the nonparametric (Mann Whitney U) test. Trials were considered significantly different when p<0.05. The cut-off value was used to determine the positivity criteria of the test, which was determined by analyzing the ROC (Receiver Operating Characteristic) curve used to evaluate the sensitivity and specificity of the assay.

## Results

### Construction, Expression and Purification of the Recombinant Fusion Protein from *Mycobacterium tuberculosis*

To construct the recombinant CMX fusion protein (C from Ag85C, M from MPT-51 and X from HspX) containing epitopes from *Mtb* Ag85C, MPT51 and HspX, an expression plasmid encoding the 6× His-tagged pET23a/CMX fusion as a single protein under the control of T7 promoter was constructed by PCR amplification and subsequent cloning of genes into *BamHI*/*HindIII* sites of the pET23a plasmid (Fig. S1 and S2). The strategy also incorporated a flexible linker between each protein.

**Figure 1:**
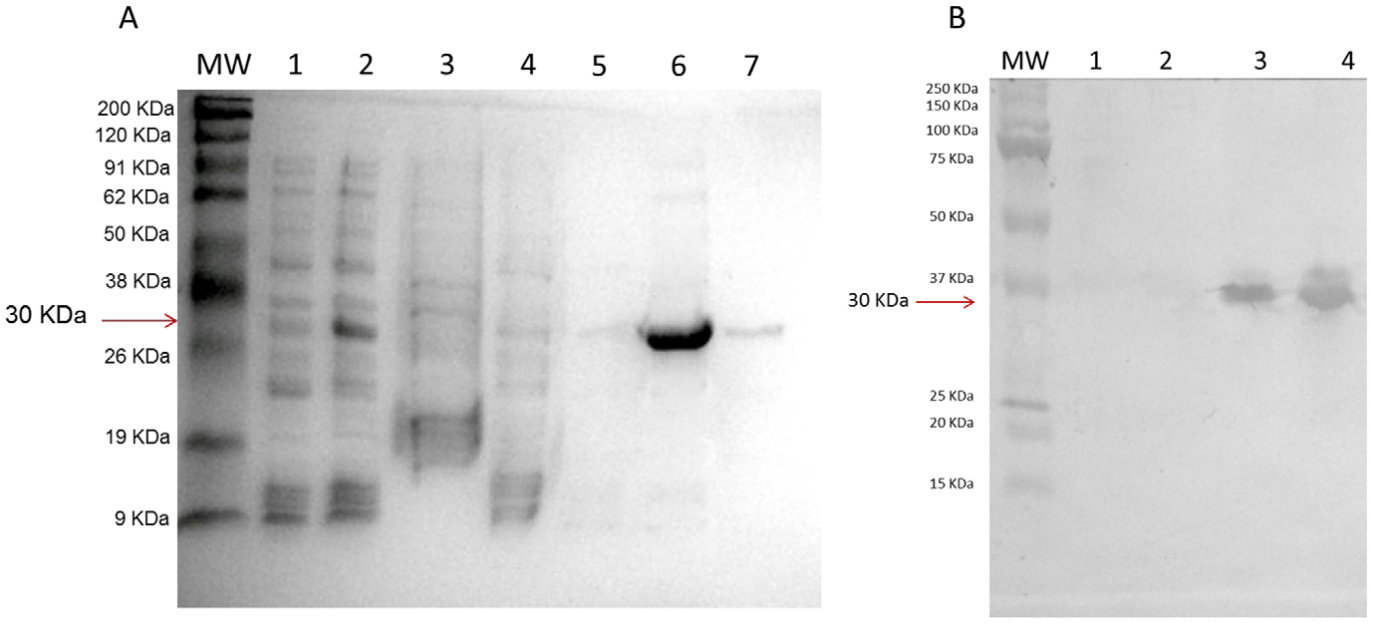
Purification and expression of the recombinant Ag85C-MPT51-HspX (CMX) **fusion protein.** (A) Purification under denaturing conditions and analysis of the recombinant CMX protein by 12% SDS-PAGE stained with Coomassie Blue. MW: molecular weight marker. Lane 1: cell lysate fraction of the column before application (without IPTG); lane 2: cell lysate fraction of the column before application (with IPTG); lane 3, the supernatant fraction before application onto the column; lane 4, the binding fraction after application on the column; lane 5, the wash fraction after application on the column; lane 6 and 7, fractions of the eluted recombinant protein of approximately 30 kDa. Each lane contains the equivalent of 10 µl of each fraction. (B) The expression of the recombinant CMX protein was analyzed by immunoblot using anti his tag-HRP. MW: molecular weight marker. Lane 1: Cell lysate containing pET23a without IPTG; lane 2: cell lysate containing pET23a with IPTG; lane 3: cell lysate containing pET23a/CMX without IPTG; lane 4: cell lysate containing pET23a/CMX with IPTG.

The gene corresponding to the fusion protein was cloned, sequenced, and expressed in *Escherichia coli* BL21 (DE3). The recombinant protein was purified by affinity chromatography using Ni-NTA affinity columns. The purified recombinant protein was analyzed by SDS-PAGE. The protein had the expected size of ∼30 kDa (Fig. 1A), and the expression was confirmed by immunoblot analysis using an anti-his tag monoclonal antibody (Fig. 1B), which confirmed the correct expression of the recombinant fusion protein.

### The Liposome-encapsulated CMX Fusion Protein Vaccine is Antigenic and Immunogenic to Mice

To evaluate whether CMX could be used as a subunit vaccine, vaccines were formulated using liposome encapsulation and CpG DNA as an adjuvant. BALB/c mice received three subcutaneous immunizations with CMX and CpG DNA. The control groups were vaccinated with only liposome-encapsulated CpG DNA, liposome alone, or saline.

**Figure 2:**
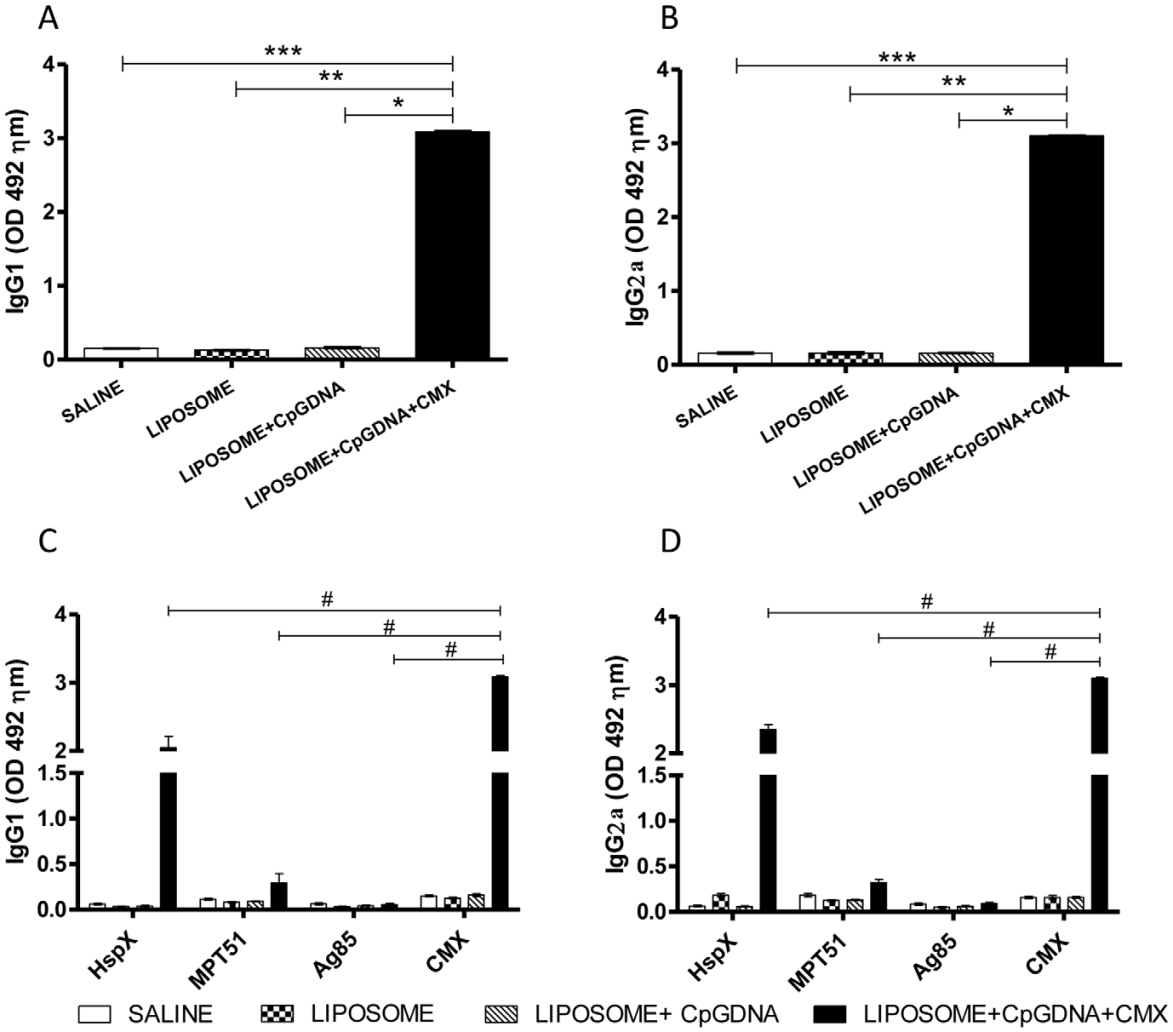
Production of antibodies against the anti-fusion protein Ag85C-MPT51-HspX (CMX) antibodies. Mice were immunized three times with the CMX fusion protein; 15 days after the final immunization, serum samples were collected from the mice, and the levels of IgG1 (A) and IgG2a (B) antibodies were evaluated. A and C show the absorbance from IgG1 specific responses and B and D show the absorbance for IgG2a. The results show the means and standard deviations of five animals per group. Statistically differences: * Comparison between the group immunized with CMX vaccine formulation and the group immunized with CpG DNA formulation; ** Comparison between the group immunized with CMX vaccine formulation and the group immunized with liposome; *** Comparison between the group immunized with CMX vaccine formulation and the group immunized with saline. # Response to CMX compared to other antigens. This experiment was independently repeated with similar results.

The IgG1 and IgG2a antibody levels were measured fifteen days after vaccination. Higher levels of IgG1 (3.08±0.04) and IgG2a (3.10±0.03) were observed in mice from the vaccinated group (Fig. 2). Thus, the CMX vaccine formulation was able to induce a specific humoral immune response in BALB/c mice.

**Figure 3:**
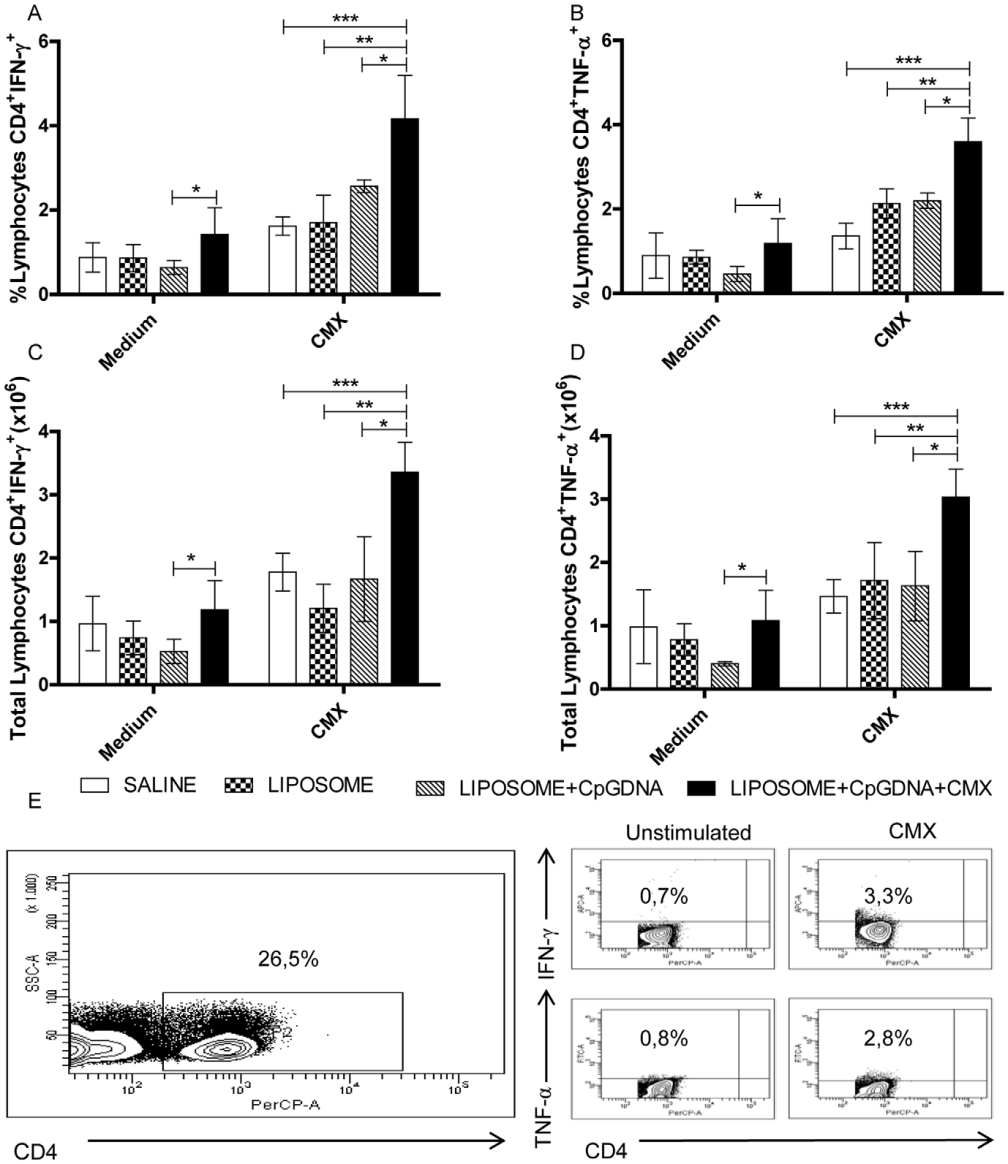
Induction of Th1 cytokines after immunization with the Ag85C-MPT51-HspX (CMX) fusion protein. Groups of mice were immunized three times, 15 days apart, by subcutaneous administration of CMX vaccine formulation, CpG DNA liposome-encapsulated, liposome alone or saline. Four weeks after the last immunization splenocytes were stimulated with CMX protein prior to flow cytometry acquisition. The frequency (A and B) and total number (C and D) of T CD4^+^ lymphocytes expressing IFN-γ^+^, (A and C) or TNF-α^+^ (B and D) are demonstrated. Dot plots representing the gating settings are shown in (E), demonstrating the CD4^+^ lymphocytes gating and its expression of IFN-γ^+^ and TNF-α^+^. The results represent the means and standard deviations of five animals per group. Statistically differences: * Comparison between the group immunized with CMX vaccine formulation and the group immunized with CpG DNA formulation; ** Comparison between the group immunized with CMX vaccine formulation and the group immunized with liposome; *** Comparison between the group immunized with CMX vaccine formulation and the group immunized with saline. This experiment was independently repeated with similar results.

To determine whether a Th1-specific immune response was induced, TCD4^+^ splenocytes were analyzed by flow cytometry. The percentage of TCD4^+^ cells expressing IFN-γ (Fig. 3A and 3E) in the group of mice immunized with CMX was greater than the percentage of these cells in the group of mice immunized with liposome-encapsulated CpG DNA, liposome alone or saline. Similarly, higher percentages of TCD4^+^ cells expressing TNF-α (Fig. 3B and 3E) were observed in the group of mice immunized with CMX when compared to the percentage of TCD4^+^TNF-α^+^ cells in the groups of mice immunized with liposome-encapsulated CpG DNA, liposome alone or saline. The same differences remained when total numbers of cells were analyzed (Fig. 3C and 3D). These results suggested that the vaccine formulation containing the CMX protein was able to induce a TCD4-specific immune response.

### CMX is Recognized Essentially by Antibodies from Patients with Active TB

The CMX protein is not naturally produced by Mtb but contains portions of three distinct proteins (Ag85C, MPT51 and HspX) that have individually been shown to induce a specific immune response in TB patients. Therefore, we asked whether the recombinant protein maintained the ability to be recognized by sera from patients with active TB. An ELISA was optimized using the fusion protein, and serum samples from patients with active TB or healthy controls individuals were assessed.

The study population was mainly composed of male patients and 73.5% were vaccinated with BCG, as shown in Table 1. Of the participants, 88.6% were sputum AFB positive. In the control group, 51% of the individuals were vaccinated with BCG, and all had negative tuberculin skin test (TST less than 10 mm). The groups were matched according to sex and age. The number of individuals with TB and healthy controls comprising the study population was determined based on the incidence of TB in the region of Goiânia, Goias, Brazil.

**Figure 4:**
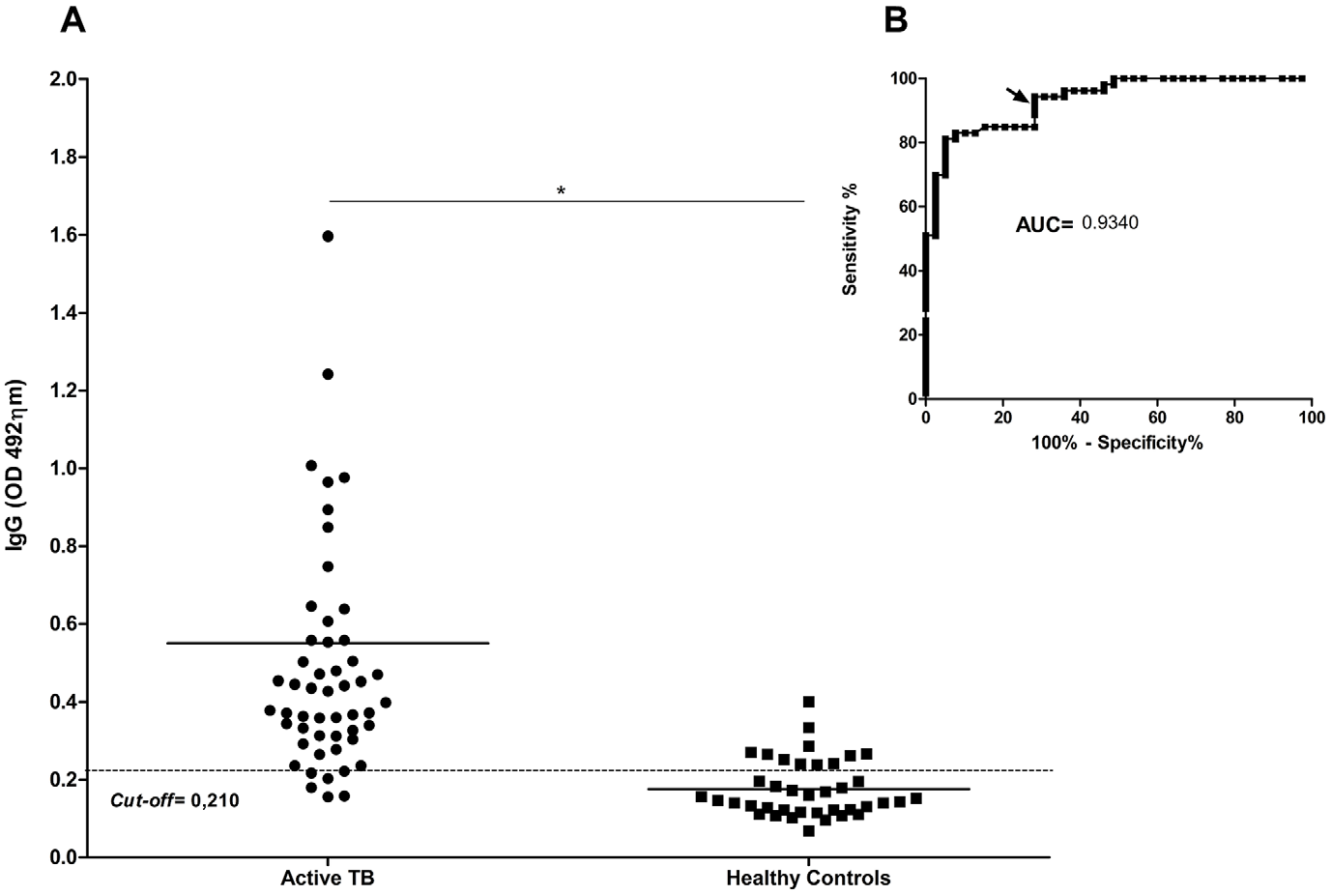
Analysis of the IgG antibody response to the Ag85C-MPT-51-HspX fusion protein in individuals with active TB and healthy controls. Each dot represents one patient or control. (A) The solid lines in each group indicate the median value. The dotted line indicates the *cut-off* value, which was determined by ROC Curve analysis; the *cut off* is identified by the arrowhead on B inset. The P-values of the differences in absorbances between the groups, indicated above the plots, was determined by t-tests.

Individuals with active TB had higher serum levels of IgGs specific to CMX (0.776±0.309) compared to healthy controls (0.373±0.271) (p<0.0001) (Fig. 4). The receiver operating characteristic (ROC) curve describing the relationship between the sensitivity and specificity at any cut-off value is also presented, and the area under the curve (AUC) was 0.934. Based on the AUC value and an adopted cut-off value of 0.210, the specificity was 71.79% (CI = 95%; 55.13% to 85.0%), and the sensitivity was 92.45% (CI = 95%; 81.79% to 97.91%). The analysis of the accuracy of the ELISA test found a positive predictive value (PPV) of 81.6% and a negative predictive value (NPV) of 87.5%.

**Figure 5:**
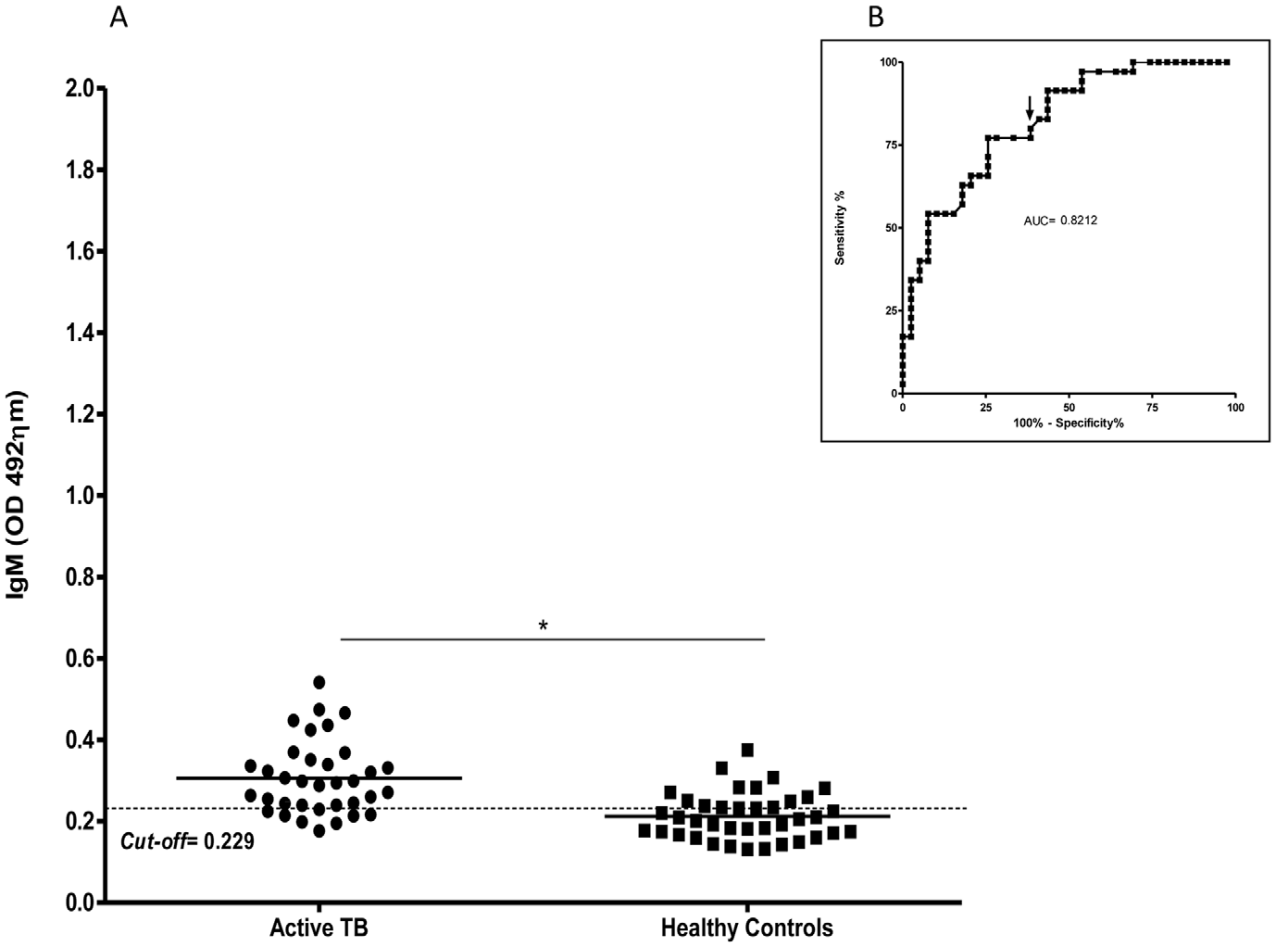
Analysis of the IgM antibody response to the Ag85C-MPT-51-HspX fusion protein in individuals with active TB and healthy controls. Each dot represents one patient or control. (A) The solid line on each group indicate the median values. The dotted line indicates the *cut-off* value, which was determined by ROC Curve analysis; the *cut off* is identified by arrowhead on B inset. The P-value of the differences in absorbances between the groups, indicated above the plots, was determined by t-tests.

The analysis of specific IgM serum antibody levels in individuals with active TB also revealed an increase (0.305±0.09) when compared to the healthy controls (0.212±0057; p<0.0001) (Fig. 5). The area under de curve was 0.82, and an arbitrary cut-off was established at 0.229. The specificity for the ELISA was 61.54%, and the sensitivity was 80.0%. This test showed a PPV of 65.0% and an NPV of 77.0%. Thus, the ELISA results presented here (Fig. 5 and 5) demonstrate that the humoral immune response to the CMX protein could discriminate between patients with active TB and healthy controls.

## Discussion

To control the spread of TB and its MDR/XDR-TB forms is one of the greatest public health challenges. This may be achieved by combining multiple strategies, such as by developing more accurate diagnostic tests to differentiate between TB and latent TB infection, especially in endemic areas, or developing new vaccines that confer effective protection by improving the protection provided by BCG, and finally by developing new anti-mycobacterial drugs. In this study, we constructed a fusion protein incorporating the Mtb antigens Ag85C, MPT51 and HspX, which are expressed at different stages during TB infection and are consequently recognized at diverse stages of the human disease. We have demonstrated here that our protein construct continued to be immunogenic in mice and could be distinctly recognized by individuals with active TB, suggesting the successful expression of the recombinant protein, most likely preserving the original antigenic epitopes.

The Th1 immune response is associated with the induction of specific IgG2a antibodies due to the IFN-y production, while the induction of the Th2 immune (mainly IL-4/IL-10) response is associated with IgG1 antibodies [24]–[25]. To examine the immune response elicited by the vaccine formulation containing the CMX protein, the levels of the different subclasses of antibodies were analyzed. Higher levels of IgG1 (3.08±0.04) and IgG2a (3.10±0.03) antibodies specific to the CMX protein were observed (Fig. 2). These antibody classes were similarly induced to levels comparable to those induced by other fusion protein constructs, such as the Ag85B-MPT64_190–198_-HspX construct that was used as vaccine booster for BCG in mice or Mtb10.4-HspX (MH) emulsified in DDA–TDM for use as subunit vaccine [26]–[27].

The importance of antibodies in Mtb Infection is still controversial; however, during the extracellular phase, specific antibodies can bind to mycobacteria, facilitating absorption and phagocytosis [28]. In addition, IgG2a, followed by and to a lesser degree IgG1, antibodies increase the microbicidal activity of macrophages, NK cells and cytotoxic T cells by ADCC [24]–[29]–[30].

The CMX fusion protein was tested in a vaccine formulation, and here it was demonstrated that IFN-γ (4.16%) and TNF-α (3.6%) specific TCD4 positive cells were induced (Fig. 3). The TCD4 cells play a central role in TB-induced immunity [31] by producing IFN-γ and TNF-α, which activate the macrophages that play an important role in the initial immune response against Mtb, including the formation of the granuloma [32]–[33]. IFN-γ and TNF-α synergistically enhance the microbicidal mechanisms of murine macrophages *in vitro* [34].

Studies have provided convincing evidence that the protection against TB in humans and mice mainly depends on the Th1 response and the protective cytokines IFN-γ, IL-17 and TNF-α [35]. Strategies to enhance the immunogenicity of a vaccine, such as the use of CpG (a pathogen associated molecular pattern, PAMP), allows cells of the innate immune system to be activated via TLR-9, a pattern recognition receptor (PRR) that induces preferentially a Th1 immune response [36]–[37]. Additionally, a good delivery system can dictate the success or failure of a vaccine candidate. Here, liposomes were used to improve antigen delivery and presentation to B and T cells [38]–[39]–[40].

Several studies describing the development of new subunit vaccines against TB suggest that for a protein to be considered a good subunit candidate vaccine, it must first induce pro-inflammatory cytokines and specific antibodies before other groups of subset T cells or cytokines are evaluated. The immunogenicity of the CMX fusion protein was demonstrated in mice by the elicitation of Th1 cytokine production (IFN-γ and TNF-α) by splenocytes and the induction of specific antibodies in mice immunized with the CMX protein. The results indicate that the CMX fusion protein was immunogenic, and although only the antibody responses and cytokine production were assayed, the data show that the CMX protein could significantly induce Th1 and Th2 immunity that may act specifically against TB infection.

Our results are in agreement with others that also tested fusion proteins, as a vaccine candidate for TB, although in our assay, the protective efficacy was not tested. Bertholet et al [41] characterized the immunogenicity of ID93, a fusion protein containing Rv3619, Rv1813, Rv3620, and Rv2608, with the adjuvant GLA-SE in mice and demonstrated that the combined induction of polyfunctional CD4 Th1 cells producing specific IFN-γ, TNF-α and IL-2 was able to reduce the number of bacteria in the lungs of animals infected with Mtb and MDR-TB. Niu et al [27] evaluated the immunogenicity of an MH fusion protein (Mtb10.4-HspX) with DDA–TDM as adjuvant in mice and observed the induction of a humoral immune response and cell-mediated immunity [27].

Previous studies have shown that immunization of mice with the combination of Ag85A and HspX enhanced the protective effect in the acute phase and chronic TB infection [42]. Similarly, Uto et al. [43] showed in mice that a fusion protein containing the MPT51 and Hsp70 antigens in a subunit vaccine induced an immune response of CD4 T cells producing IFN-γ in response to *in vivo* MPT51 antigen stimulation. Thus, we believe our vaccine has good potential use in a protection assay, and we are currently addressing this issue.

To further validate the immunogenic properties of the CMX fusion protein, further studies are required to evaluate the subset of T cells involved and the ability to generate memory cell populations to produce regulatory cytokines as well as other inflammatory mediators, and most importantly challenge with *M. tuberculosis* to address the vaccine efficacy.

Because CMX was shown to be immunogenic in mice, the next step was to investigate the usefulness of CMX in the diagnosis of human TB. To determine the ability of the CMX fusion protein to distinguish active TB, we evaluated the humoral immune response of individuals from a TB-endemic area, where most of the population had been vaccinated with BCG and most likely had come in contact with other environmental mycobacteria.

In the search for new antigens for the diagnosis of TB, it has been shown that IgG antibodies specific to the HspX antigen were not able to distinguish between patients with tuberculosis and healthy controls (sensitivity of 60.8%) compared to other proteins produced and tested in TB serological tests (such as HspX alone, sensitivity of 67.3%; or a mixture of ESAT-6, CFP10 and antigen 85A, sensitivity of 77.7%) [44]. Here, when a fusion combining three different proteins was used, the levels of specific IgG measured by ELISA improved the sensitivity to 92.45%, overcoming the WHO goals [45], which determine that the sensitivity of a diagnostic test for TB must be above 80%. Furthermore, previous work evaluating the ability of IgM antibodies specific to MPT51 to distinguish patients with active TB from healthy controls revealed that this antigen was promising (p<0.001); however, the sensitivity of this assay was 67.3% [11]. IgG antibodies specific to MPT51, however, were not able to discriminate between patients with active TB and healthy controls [11]. Again, in this study, IgG antibodies against the CMX fusion protein efficiently discriminated between the two groups (p<0.0001).

Due to its immune dominance, Ag85C has been rated as a strong candidate marker for the diagnosis of tuberculosis [46]–[48]. Previous work using antigen 85C for TB serodiagnosis showed a good discrimination between individuals with TB and healthy controls, where 80% of the TB patients were responsive to the test [48]. Using the immunodominant fragments of Ag85C, MPT51, and HspX proteins, in the CMX fusion protein, improved the recognition by active TB patients, although we could not directly compare our data with the results obtained with Ag85 alone or in a different TB endemic setting. The results of this study allowed us to achieve improved sensitivity, specificity and accuracy (ROC with an area under the curve of 0.9), that seems better than other studies using fusion proteins for TB diagnosis, though a direct comparison cannot be made [44]–[48]–[49]–[50]. Future work, using increased number of active TB individuals both sputum AFB negative and positive, may show the importance of ours or other ELISAs to help to improve the TB diagnosis. To validate the effectiveness of the CMX fusion protein in the serodiagnosis of TB infection, another sampling of the endemic area is necessary.

Surprisingly, in an analysis of the cost and effectiveness of TB serological diagnostic tests, Menzies et al. reported that most of the serological diagnostic tests for TB in India presented false positive and false negative results, representing a social and financial risk [51]. We believe that a serological test would be a financial risk if the health authorities did not assume its cost. Moreover, instead of condemning these tests, one should address the usefulness of them in a different population to test for the effectiveness.

Despite the complexity of the cellular and humoral immune response induced by Mtb, serological markers to distinguish between healthy and sick individuals remains attractive to researchers around the world [52]. The known protective immune response against TB is the predominant T cell response. However, TB patients produce antibodies to Mtb proteins, so there is still much to be explored in terms of the relationship between the production of antibodies, the antibody specificity and the disease progression [53]. Several diagnostic tests based on the cellular immune response have been developed, reducing the problem of cross-reactivity with environmental mycobacteria, BCG vaccination and even latent TB infection. However, these tests do not discriminate between LTBI in healthy individuals who had TB and were cured [54]. Furthermore those tests are expensive for any country that presents estimates of over 70,000 TB cases per year, such as Brazil. Thus, a serological test continues to be a goal, although the ideal test has not been yet been developed.

An ideal screening test for TB should have the ability to identify individuals with latent TB infection that can develop active TB and consequently, direct those individuals to perform an IFN-γ release assay so that other respiratory diseases can be ruled out. A test based on serodiagnosis with a high sensitivity and the predictive capability to rule out the disease is also an important tool in the diagnosis of TB, which would impact the financial resources devoted to additional tests, such as sputum, culture or X-ray, reducing the waiting time between the diagnosis and treatment initiation. Another advantage of a test based on serodiagnosis over the IGRA assay is the cost and time needed to obtain results.

In summary we demonstrated that the CMX fusion protein was shown to be immunogenic for both mice and humans. The capacity to induce both humoral and cellular specific immune response may potentiate the chances of this antigen to be used as vaccine for TB. Also we showed that ELISA using this protein was able to discriminate active TB patients from healthy controls showing a strengthen use of this protein in the development of new diagnostics tests.

## Supporting Information

Figure S1 **Schematic of the construction of the CMX expression cassette.** The first step in the construction of the recombinant protein was to amplify by polymerase chain reaction (PCR) the selected epitopes of the proteins of interest and to create specific restriction endonuclease sites. The amplicons were cloned in the pGEM-T easy vector, and each plasmid was digested with the appropriate restriction enzymes to generate a ligation mixture containing the three amplicons. The ligation mixture containing the three genes of interest was amplified using the most external primers, i.e., the amino terminal of Ag85C and the carboxy terminal of HSPX. The amplicon resulting from this amplification was inserted into the pGEM-T easy vector, digested with the enzymes *BamHI* and *HindIII*, purified, and ligated into the expression vector pET23a.
(TIFF)

Figure S2 **Map of the construction of the recombinant plasmid containing the gene for the Ag85C-MPT-51-HspX (CMX) fusion protein.** The PCR product corresponding to the fusion of the three epitopes was cloned into the pGEM-T easy vector, sequenced and subsequently transferred to the pET23a expression vector by digestion with specific restriction enzymes and cloned into the *Escherichia coli* BL21 (DE3) pLysS. The expression of the CMX fusion protein is driven by a T7 promoter, and the arrow indicates the direction of transcription.
(TIFF)

Table S1 **Primer sequences used in this study and introduced restriction sites.**
(DOCX)
