# Correction: Immunogenicity of a Fusion Protein Containing Immunodominant Epitopes of Ag85C, MPT51, and HspX from *Mycobacterium tuberculosis* in Mice and Active TB Infection

**DOI:** 10.1371/annotation/e4172d52-fc6c-40f1-bc2e-ca3df106d49f

**Published:** 2013-09-09

**Authors:** Eduardo Martins de Sousa, Adeliane Castro da Costa, Monalisa Martins Trentini, João Alves de Araújo Filho, André Kipnis, Ana Paula Junqueira-Kipnis

There were multiple errors and incorrect omissions in the Funding Statement. The Funding Statement should read: "This work was supported by the following CNPq grants: 472909/2011-8, 472906/2011-9, 301976/2011-2, 561004/2010-2, and FAPEG grants: 200910267000381, 200910267000446. EMS, MMT, and AK received fellowships from CNPq. ACC received fellowship from CAPES. The funders had no role in study design, data collection and analysis, decision to publish, or preparation of the manuscript." 

