# Supplementary figures and images for: Immunogenicity of a Fusion Protein Containing Immunodominant Epitopes of Ag85C, MPT51, and HspX from Mycobacterium tuberculosis in Mice and Active TB Infection
Source: PLoS One. 2012 Oct 25;7(10):e47781. doi: 10.1371/journal.pone.0047781 (PMC3485045; doi:10.1371/journal.pone.0047781)

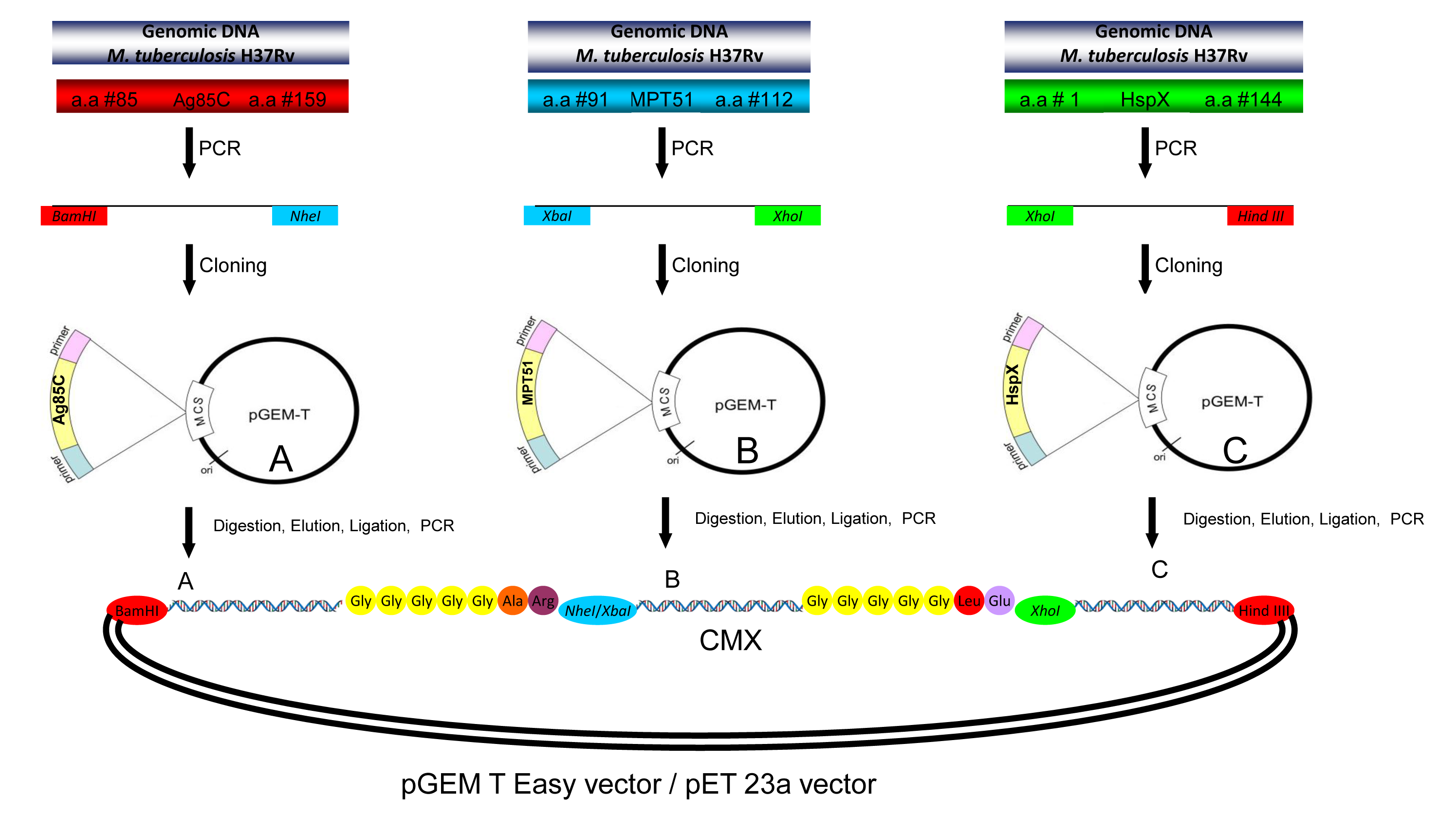

Supplement: Figure S1 — Schematic of the construction of the CMX expression cassette. The first step in the construction of the recombinant protein was to amplify by polymerase chain reaction (PCR) the selected epitopes of the proteins of interest and to create specific restriction endonuclease sites. The amplicons were cloned in the pGEM-T easy vector, and each plasmid was digested with the appropriate restriction enzymes to generate a ligation mixture containing the three amplicons. The ligation mixture containing the three genes of interest was amplified using the most external primers, i.e., the amino terminal of Ag85C and the carboxy terminal of HSPX. The amplicon resulting from this amplification was inserted into the pGEM-T easy vector, digested with the enzymes BamHI and HindIII, purified, and ligated into the expression vector pET23a. (TIFF) [file pone.0047781.s001.tiff]

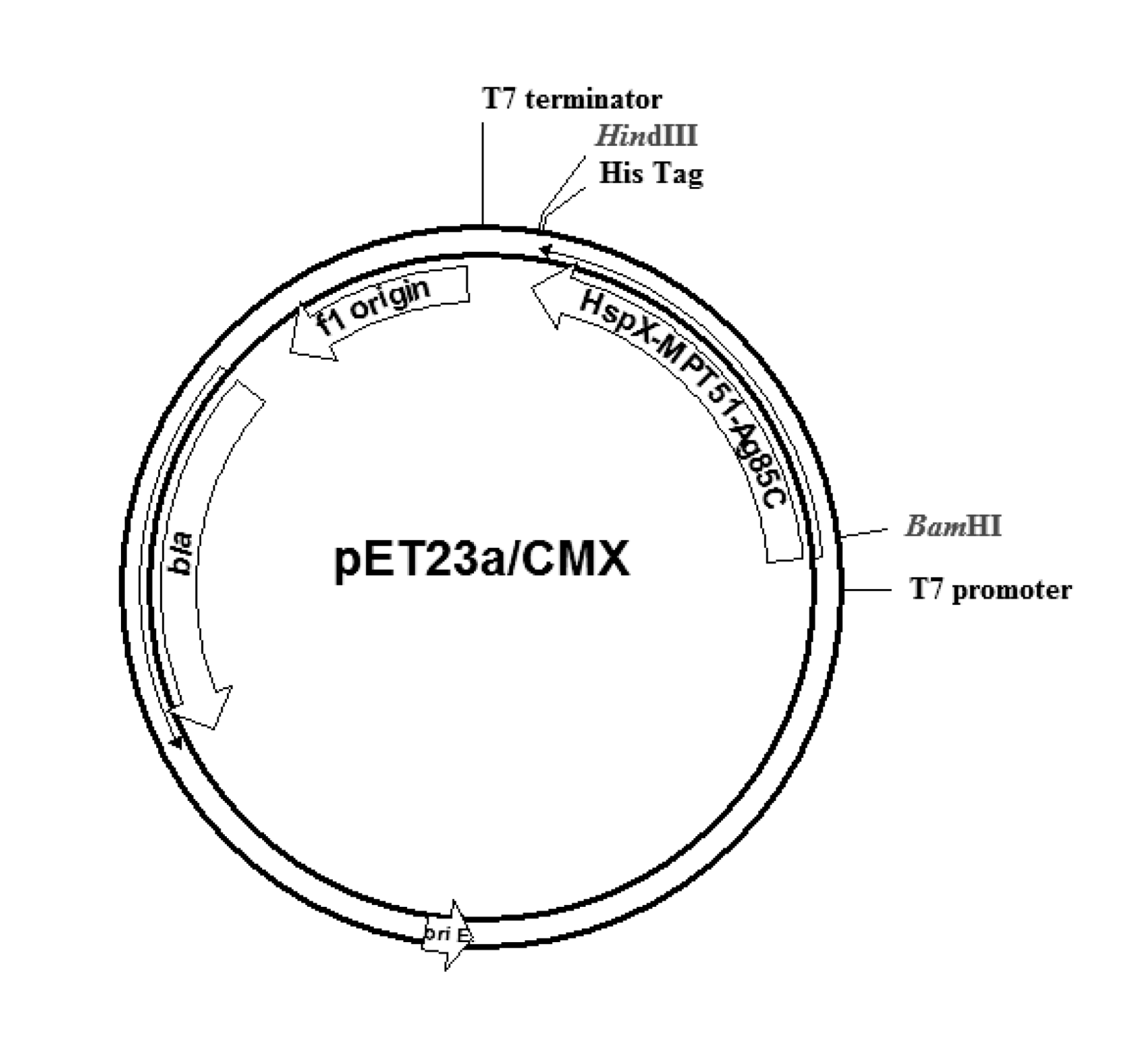

Supplement: Figure S2 — Map of the construction of the recombinant plasmid containing the gene for the Ag85C-MPT-51-HspX (CMX) fusion protein. The PCR product corresponding to the fusion of the three epitopes was cloned into the pGEM-T easy vector, sequenced and subsequently transferred to the pET23a expression vector by digestion with specific restriction enzymes and cloned into the Escherichia coli BL21 (DE3) pLysS. The expression of the CMX fusion protein is driven by a T7 promoter, and the arrow indicates the direction of transcription. (TIFF) [file pone.0047781.s002.tiff]
