# Supplementary material for: Immunogenicity of a Fusion Protein Containing Immunodominant Epitopes of Ag85C, MPT51, and HspX from Mycobacterium tuberculosis in Mice and Active TB Infection
Source: PLoS One. 2012 Oct 25;7(10):e47781. doi: 10.1371/journal.pone.0047781 (PMC3485045; doi:10.1371/journal.pone.0047781)
Supplement: Table S1 — Primer sequences used in this study and introduced restriction sites. (DOCX) [file pone.0047781.s003.docx]

**Table S1. Primer sequences used in this study and introduced restriction sites.**

| Gene Target | Primer Sequence (5´ to 3´) | Restriction Site |
| --- | --- | --- |
| Ag85C amino | tagggtacccggtctgcgggcccaggatg | *BamHI* |
| Ag85C carboxy | taggcggccgcttagttgcctgtcggggacacgcc | *NheI* |
| MPT51 amino | aggggtaccgtctagagcggtgtatctgctggacgcc | *XbaI* |
| MPT51 carboxy | taggcggccgcttactcgagttgctgttgctgttggccatcctgctcccagttggt | *XhoI* |
| HspX amino | aggggtacctctcgagatggccaccacccttcccg | *XhoI* |
| HspX carboxy | taggcggccgctcagttggtggaccggatctgaatgtg | *HindIII* |
